# Moving Abrikosov vortex lattices generate sub-40-nm magnons

**DOI:** 10.1038/s41565-025-02024-w

**Published:** 2025-10-16

**Authors:** Oleksandr V. Dobrovolskiy, Qi Wang, Denis Yu. Vodolazov, Roland Sachser, Michael Huth, Sebastian Knauer, Alexander I. Buzdin

**Affiliations:** 1 Cryogenic Quantum Electronics, Institute for Electrical Measurement Science and Fundamental Electrical Engineering, Technische Universität Braunschweig, Braunschweig, Germany; 2 Laboratory for Emerging Nanometrology, Technische Universität Braunschweig, Braunschweig, Germany; 3 Hubei Key Laboratory of Gravitation and Quantum Physics, Institute for Quantum Science and Engineering, Huazhong University of Science and Technology, Wuhan, China; 4 Physikalisches Institut, Goethe University, Frankfurt am Main, Germany; 5 Faculty of Physics, University of Vienna, Vienna, Austria; 6 Université Bordeaux, CNRS, LOMA UMR 5798, Talence, France

**Keywords:** Single photons and quantum effects, Magnetic properties and materials

## Abstract

Magnons, the quasi-particles of spin waves, are promising candidates for developing wave-based computing and hybrid quantum technologies. However, generating short-wavelength magnons through microwave excitation becomes increasingly challenging because the excitation efficiency decreases as the antenna size shrinks. Here we demonstrate an alternative approach and generate magnons in a Co–Fe strip using magnetic flux quanta, that is, Abrikosov vortices, moving in an adjacent Nb–C superconductor at velocities exceeding 1 km s^−1^. The moving vortex lattice acts on the magnetic layer via both static and dynamic stray fields. Our experiments showcase the unidirectional excitation of sub-40-nm wavelength magnons and their coherent interaction with the moving vortices. In turn, the Nb–C sustains its low-resistive state because the magnon creation removes energy from the superconductor. This discovery enables high-speed on-chip electrically driven magnon generation and validates an alternative means of magnon excitation. Our approach could be adapted to other wave excitations, such as surface acoustic waves, for integration into advanced electronic and hybrid quantum systems.

## Abstract (web summary)

In a hybrid superconductor–ferromagnet device, the dynamic stray fields of current-driven vortices unidirectionally excite coherent short-wavelength magnons.

## Main

Magnons are regarded as potential data carriers for future information-processing technologies^1^. Their properties—such as low energy dissipation and high propagation speeds—make them well suited for applications in radio frequency technology^2^ and hybrid quantum systems^3^ at the nanoscale. Magnons can carry information without relying on charge-based currents, offering a route to low-power high-frequency electronics, critical to advancing communication technologies and integration into future electronic systems^4^. The manipulation of magnons at cryogenic temperatures^5,6^ further enhances their potential as reduced thermal noise allows more efficient operation in hybrid quantum systems^7^.

One substantial challenge in utilising magnons in nanoelectronic devices is achieving sources of monochromatic magnons. Current research has concentrated on the utilization of microwave antennas, about 50–100 nm in size, to generate multiple magnons based on inductive designs. However, advancing the miniaturization of these antennas introduces notable fabrication challenges, particularly when the dimensions of the antennas become smaller than the electron mean free path. Additional complications arising from these reduced dimensions include a rapid increase in resistivity, inadequate impedance matching and heightened microwave losses, all resulting in diminished excitation efficiencies. Moreover, inductive antennas emit radiation in multiple directions.

To achieve unidirectional excitation alongside efficient and low-dissipative magnon generation, theoretical proposals suggested utilizing different fast-moving magnetic sources to perturb magnetic moments, including moving magnetic monopoles^8,9^, domain walls^10–13^ and Abrikosov vortices^14–17^. Until now, fast enough moving magnetic sources have not been realized, while recent studies^18^ suggest that fast-moving magnons can be generated using optical pulses. However, at the required high-magnon velocities (a few kilometres per second in ferromagnet-based devices), domain walls collapse because of Walker breakdown^19^, and the lack of long-range order in vortex arrays^20^ makes in-phase generation of magnons hardly feasible for most superconductor-based systems. We note that domain walls can move notably faster in ferrimagnets^21^, antiferromagnets and ferromagnetic nanotubes. However, the immunity of antiferromagnets to magnetic fields poses substantial challenges in manipulating domain walls. Ferrimagnets must operate near the angular momentum compensation temperature^22^, while high-quality round ferromagnetic nanotubes with sufficiently low damping remain inaccessible so far^23^.

Here, we experimentally demonstrate the generation of short-wavelength magnons in a Co–Fe magnonic conduit initiated by a lattice of fast-moving magnetic flux quanta (Abrikosov vortices) in an adjacent Nb–C superconducting strip. Using a microwave ladder antenna, we observe sub-40-nm wavelength magnons travelling over a distance of about 2 μm through the magnonic conduit. In addition, we find that magnon generation induces a constant-voltage Shapiro step across the superconducting strip. This magnon Shapiro step emerges because of the phase-locking of the vortex lattice with the excited magnons, which limits the vortex velocity and represents a dynamic pinning mechanism for the reduction of dissipation in superconductor-based heterostructures^14–16^. We demonstrate that the magnon excitation is unidirectional (magnon propagates in the direction of motion of the vortex lattice) and monochromatic (magnon wavelength is equal to the vortex lattice parameter). In our experiments, we have achieved monochromatic excitation of spin waves with a wavelength of below 40 nm.

## Experimental set up and current–voltage curves

**Fig. 1:**
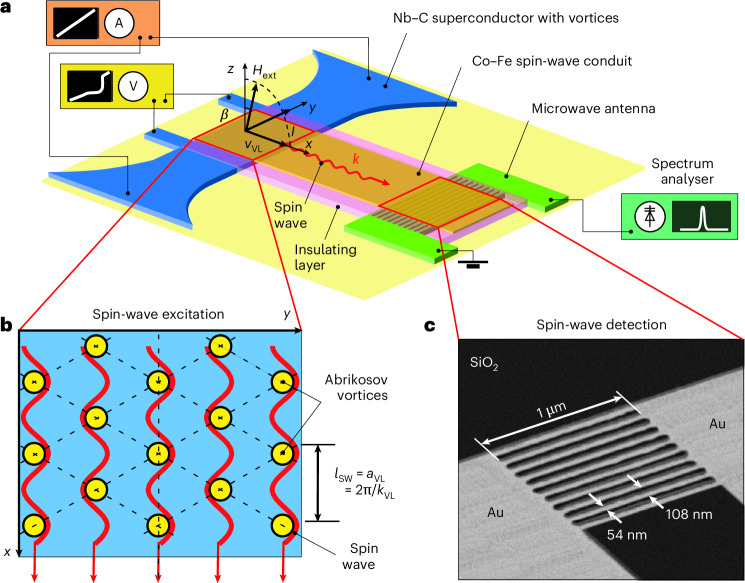
Experimental set up. **a**, The experimental geometry. The superconductor/ferromagnet hybrid structure consists of a Nb–C superconducting strip, stray field coupled to a Co–Fe magnonic conduit. Measurements are taken at 4.2 K in a magnetic field *H*_ext_ directed at a small-angle ${\beta}\approx{5}^{\circ}$ concerning the *z* axis in the *x–z* plane. **b**, A schematic of the excitation of magnons by the moving vortex lattice. **c**, A scanning electron microscopy image of the microwave ladder antenna, before the deposition of the Co–Fe conduit, used for the detection of magnons with wavelengths ${\lambda}_{\rm{SW}}\approx36{\rm{nm}}$ (wavevectors *k*_SW_ ≈ 175 rad ${{{\upmu}}{\rm{m}}}^{-1}$).

We investigate a hybrid structure consisting of a 45-nm-thick Nb–C superconducting strip and a 30-nm-thick Co–Fe ferromagnetic magnonic conduit (Fig. 1a), separated from each other by a 3-nm-thick insulating Nb–C layer and interacting through magnetic stray fields^24,25^. We take measurements at 4.2 K in the vortex state of Nb–C, below its superconducting transition temperature *T*_c_ = 5.6 K (ref. ^26^). In an external magnetic field, Nb–C is penetrated by a lattice of Abrikosov vortices (fluxons), each of which carries one quantum of magnetic flux ${\varPhi}_{0}\,\simeq\,2.068 \times10^{-15}$ Wb (ref. ^27^). We tune the vortex lattice parameter ${a}_{{\rm{VL}}}={[(2/\sqrt{3})({\varPhi }_{0}/{H}_{{\rm{ext}}})]}^{1/2}$ by varying the external magnetic field value *H*_ext_. The lattice of vortices is characterized by a modulation of the local magnetic field, which attains a maximum at the vortex cores^28^. We apply a d.c. current (*I*) in the *y* direction, which exerts on the lattice of Abrikosov vortices a Lorentz force acting in the *x* direction (Fig. 1a). At sufficiently large currents, the vortex lattice moves and induces oscillations of the local magnetic field at a given point in space at the washboard frequency *f*_VL_ = *v*_VL_/*a*_VL_.

We apply *H*_ext_ at a small tilt angle ${\beta}\approx{5}^{\circ}$ to the sample normal in the *x–z* plane (Fig. 1a), to maximize the efficiency of magnon excitation and detection (Supplementary Note 1 and Supplementary Fig. 4). The *H*_ext_ values, varied between 1.75 T and 1.95 T, are sufficient to magnetize the Co–Fe magnonic conduit to saturation, setting the magnon propagation to the quasi-forward volume spin-wave geometry^29^. The motion of vortices in the superconducting strip triggers a precession of spins in the magnonic conduit (Fig. 1b). Once the velocity of the vortex lattice in the superconductor reaches the phase velocity of magnons in the ferromagnet, the magnon generation condition is satisfied^14–16^. We detect the propagation of the excited magnons through the magnonic conduit by a spectrum analyser connected to a microwave ladder nano-antenna located at a distance of 2 μm away from the Nb–C/Co–Fe hybrid region (Fig. 1c). The excitation and detection efficiency of the antenna is illustrated in Supplementary Fig. 1.

**Fig. 2:**
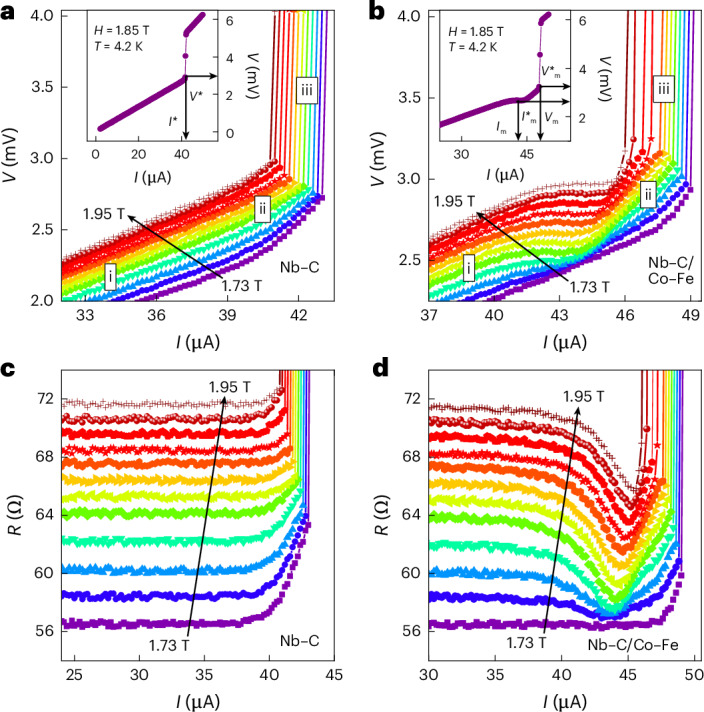
Current–voltage (*I*–*V*) curves of the samples. **a**,**b**, The Nb–C superconductor (**a**) and Nb–C/Co–Fe hybrid structure (**b**). As indicated, the magnetic field *H*_ext_ increases in steps of 20 mT from 1.73 T to 1.95 T. As the current increases, the *I*–*V* curves exhibit the regimes of flux flow (i), nonlinear conductivity (ii) and flux-flow instability (iii). The definition of the instability current $I^{*}$ and the instability voltage $V^{*}$ for the Nb–C superconductor and the respective quantities for the Nb–C/Co–Fe hybrid structure, ${I}_{{\rm{m}}}^{* }$ and ${V}_{{\rm{m}}}^{\;*}$, are indicated in the insets. The *I*–*V* curves for the Nb–C/Co–Fe hybrid structure exhibit voltage steps, with the current *I*_m_ and the voltage *V*_m_ defined at the step centre. **c**,**d**, Evolution of the electrical resistance for the Nb–C superconductor (**c**) and Nb–C/Co–Fe hybrid structure (**d**) as a function of the transport current.

First, we characterized the sample by standard d.c. current–voltage (*I–V*) measurements (Fig. 2). The motion of vortices in the superconducting strip is associated with a retarded recovery of the superconducting order parameter^28^, resulting in an ohmic branch in the current–voltage curve. This behaviour is shown in Fig. 2a for the superconducting Nb–C strip before the deposition of the magnonic conduit. Namely, the *I*–*V* curves for the bare Nb–C strip exhibit a nearly linear regime of flux flow (Fig. 2a(i)) up to a current of about 40 μA. At larger currents, the *I*–*V* curves show a regime of nonlinear conductivity (Fig. 2a(ii)) preceding the regime of Larkin–Ovchinnikov instability (Fig. 2a(iii))^30,31^. The flux-flow instability occurs at the instability current $I^{*}$, associated with the instability voltage $V^{*}$, whose definition is shown in the inset of Fig. 2a.

Distinct from the bare Nb–C strip, in the *I*–*V* curves for the Nb–C/Co–Fe hybrid structure, we observe constant-voltage steps, occurring at voltages of *V*_m_ (Fig. 2b). These voltage steps are accompanied by an expansion of the low-resistive regime towards larger currents and an increase in the instability current, ${I}_{{\rm{m}}}^{* } > {I}^{* }$, and the instability voltage, ${V}_{{\rm{m}}}^{* } > {V}^{* }$, for the Nb–C/Co–Fe structure in comparison with the bare Nb–C (Fig. 2c,d). From the instability voltage, we deduce the maximal vortex velocity by the standard relation $v^{*}=V^{*}/(H_{\rm{ext}}L)$ (ref. ^28^), where *L* = 1 μm is the distance between the voltage leads.

## Microwave detection of magnons

**Fig. 3:**
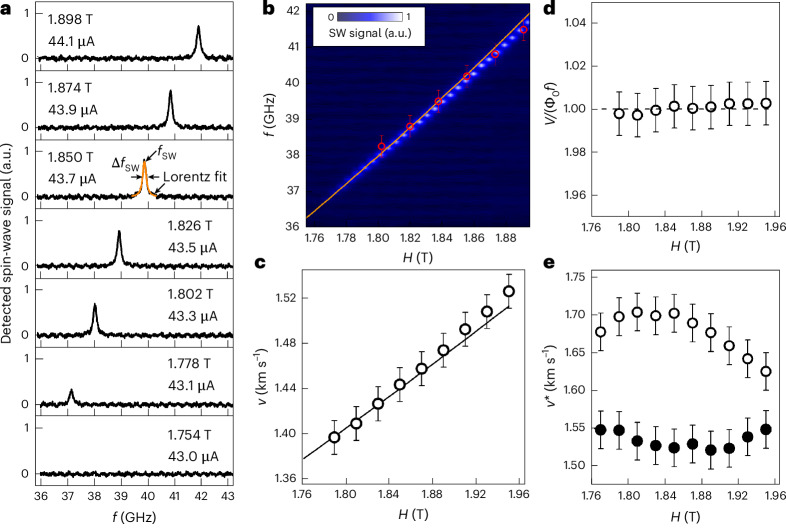
Microwave detection of propagating magnons. **a**, Normalized detected magnon frequency spectra for a series of magnetic field and current values. **b**, Normalized detected spin-wave signal versus frequency and magnetic field value. The open circles show the washboard frequency of the vortex lattice, *f*_VL_ = *v*_VL_/*a*_VL_, as deduced at the centre of the voltage step from the *I*–*V* curves. The solid line shows the spin-wave frequency *f*_SW_ at the magnon generation condition, as deduced from the micromagnetic simulations. **c**, Vortex velocity *v*_VL_ (symbols) deduced at the centre of the voltage step in the *I*–*V* curves in comparison with the phase velocity of magnons *v*_SW_ (solid line) at the magnon generation condition, as deduced from the micromagnetic simulations. **d**, Normalized step voltage *V*_m_ (symbols), as deduced from the *I*–*V* curves, in comparison with the Shapiro step voltage given by $V/({\varPhi}_{0}fN)=1$ (dashed line). **e**, Flux-flow instability velocity $v^{*}$ as a function of the magnetic field value for the bare Nb–C strip (solid symbols) and the Nb–C/Co–Fe hybrid structure (open symbols). In **b**–**e**, the error bars are the s.e.m.

For the detection of magnons excited by moving vortices, we tune the transport current *I* to *I*_m_, which corresponds to the middle of the voltage steps in the *I*–*V* curves (Fig. 2b). The detected microwave signals exhibit peaks at the frequency *f*_SW_, which increases with increasing *H*_ext_ (Fig. 3a). We note that we detected no microwave signal for the bare Nb–C strip. Thus, the detected signal must stem from the spin-wave excitation in the magnonic conduit rather than being directly emitted from vortices^14,32^. Also, we detected no microwave signal for the current applied in the −*y* direction, resulting in the vortex motion away from the antenna (−*x* direction), as shown in Supplementary Fig. 2d. This finding suggests that the spin-wave excitation is unidirectional.

We observed a linear dependence of the detected magnon signal’s frequency on the magnetic field value *f*_SW_(*H*_ext_) (Fig. 3b). We have also revealed that the detected peak frequency *f*_SW_ matches, within 5% accuracy, the washboard frequency of the vortex lattice *f*_VL_. We have established that the magnetic field dependence of the vortex velocity, *v*_VL_(*H*_ext_), deduced from the voltage step in the *I*–*V* curves, is also nearly linear (Fig. 3c). From the step voltages *V*_m_, we deduced, by the standard relation *v*_m_ = *V*_m_/(*H*_ext_*L*), that the steps occur at the vortex velocities *v*_m_ between 1.38 km s^−1^ and 1.52 km s^−1^. These velocities are about 50 m s^−1^ smaller than the typical instability velocities $v^{*}$ in the bare Nb–C superconductor, and they are approximately 200 m s^−1^ smaller than ${v}_{{\rm{m}}}^{* }$ when the superconducting Nb–C strip is overlaid with a Co–Fe magnonic conduit.

We found a noteworthy correlation between the step voltage *V*_m_ in the *I*–*V* curves (Fig. 2b) and the peak frequencies *f*_SW_ in the detected microwave signals (Fig. 3a). The magnetic field dependence of the normalized step voltage $V_{m}/({\varphi}_{0}f_{\rm{SW}}N)$ reveals that the step voltage is constant and is equal to unity (Fig. 3d). Here, *N* is the number of vortices between the voltage leads. This finding points to a fundamental nature of the voltage step, allowing us to describe our findings in terms of coherent fluxon–magnon interaction and interpret the observed voltage steps as magnon Shapiro steps^33,34^. Finally, we reveal that the vortices in the Nb–C/Co–Fe hybrid structure can reach higher velocities than in the bare Nb–C strip (Fig. 3d). Indeed, ${v}_{{\rm{m}}}^{* }$ exceeds 1.62 km s^−1^ for Nb–C/Co–Fe, while $v^{*}$ does not exceed 1.56 km s^−1^. The preservation of the low-resistive state suggests that energy is extracted from the moving vortices and transferred to excite spin waves in the adjacent ferromagnet, in line with the theoretical predictions^14–16^.

## Magnon generation by a lattice of fluxons

Before interpreting our findings in terms of magnon generation by moving fluxons, we need to rule out alternative explanations of the observed expansion of the low-resistive regime in the *I*–*V* curves of Nb–C overlaid by the Co–Fe layer. In general, adding a conducting layer close to a superconductor introduces an extra channel for the relaxation of quasi-particles (unpaired electrons) and heat removal, influencing the instability parameters through eddy currents. Specifically, adding a conducting layer can increase^35^ or decrease $I^{*}$ and $v^{*}$ (ref. ^36^), depending on the material combination. However, the overall shape of the *I*–*V* curve remains unchanged qualitatively. By contrast, in our experiments, we observe a qualitative change in the *I*–*V* curve shape due to the appearance of a voltage step. Moreover, the electrical resistance *R*(*I*) for the bare Nb–C strip is constant in the flux-flow regime (between 25 and 37 μA) and increases monotonically with further increasing current (Fig. 2c). Contrarily, *R*(*I*) for the Nb–C/Co–Fe bilayer exhibits a minimum at the foot of the instability jump (Fig. 2d).

As another potential mechanism for the $I^{*}$ and $v^{*}$ enhancement, one could think of microwave stimulation of superconductivity^37^. However, the very short energy relaxation time in Nb–C^31^ in conjunction with strong d.c. currents and high magnetic fields of about 2 T, inducing a very dense vortex lattice, allows us to rule out this mechanism in our experiments, as detailed in Supplementary Note 3.

The observed magnon generation by moving fluxons calls for discussing the underlying coupling mechanism. Namely, a superconductor and a ferromagnet, electrically isolated by a thin, nonmagnetic insulating spacer, interact via both static and dynamic stray fields, allowing for coupling at a distance^24,25,38^. This coupling originates from the magnetic fields generated by eddy currents in the superconductor and magnetic moments in the ferromagnet. Such interaction can modify the magnon dispersion relation^24^, localize spin waves^38^ and give rise to a Bloch-like band structure in the magnon spectrum^25^.

An intriguing case of coherent fluxon–magnon interaction is expected when the wavevectors of spin waves (magnons) and the vortex lattice (fluxons) match, that is, *k*_SW_ = *k*_VL_, and their energies satisfy the condition $E({k_{\rm{SW}}})={\hslash}{v}_{\rm{VL}}k_{\rm{VL}}$ (ref. ^39^). These conditions are fulfilled when the two dispersion lines for the magnons and fluxons touch/cross with one another, allowing for strong interaction. Previously, the crossing of two dispersion curves was discussed in the context of strong magnon–phonon coupling in a platinum/yttrium–iron–garnet heterostructure^40^. In that study, a resonant enhancement of the spin Seebeck effect was observed at the touch/cross points of the magnon and phonon dispersion curves.

The generation of magnons by a fast-moving fluxon lattice can also be viewed as a magnonic analogue of the Cherenkov effect. The original Cherenkov effect is associated with the radiation of electromagnetic waves by charged particles that move faster than the speed of light in a given dielectric medium. This widespread phenomenon occurs in various physical systems, all of which adhere to a fundamental condition of energy and momentum matching, $E({k})={\hslash}vk$, the Cherenkov resonance condition^39^. In the language of Cherenkov radiation, fluxons are moving magnetic entities, and magnons are the generated excitations in the magnetically ordered medium. The relation between the Cherenkov effect for single and multiple periodically arranged moving particles is illustrated in Supplementary Fig. 10.

**Extended Data Fig. 1:**
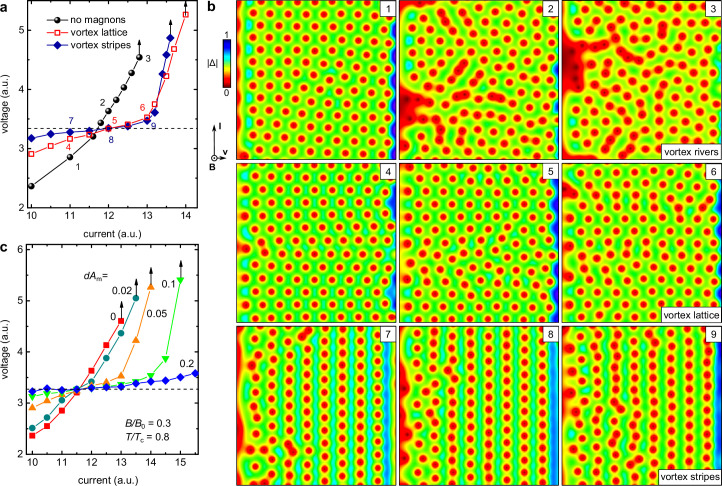
TDGL simulations of vortex dynamics upon magnon generation. **a**, *I-V* curves for a superconducting strip without magnon radiation and for two symmetries of the eddy currents (‘vortex lattice’ and ‘vortex stripes’) accompanied by a reconfiguration of the vortex lattice. **b**, Snapshots of the superconducting order parameter ∣*Δ*∣ at points 1-9 in the *I-V* curves in panel **a**. **c**, Development of the magnon Shapiro step in the *I-V* curve of the superconductor with increase of the amplitude *dA*_m_ of eddy currents upon magnon generation. With an increase in the eddy currents’ amplitude, a constant-voltage step is developed in the *I-V* curve, panel c. This is accompanied by a dynamic ordering of the vortex lattice, as concluded from the snapshots of the superconducting order parameter, panel b. Without magnon generation, the flow of vortices ordered in a nearly hexagonal vortex lattice (snapshot 1) loses its long-range order (snapshot 2) in the regime of nonlinear conductivity II. As the transport current increases, areas with a suppressed order parameter are nucleated at the edge where the vortices enter the superconductor, forming ‘vortex rivers’ (snapshot 3) and leading to an avalanche-like transition of the sample to the normal state. When the excitation of magnons is modelled with a resonance-like enhancement of the eddy currents, the *I-V* curve becomes flattened, and the motion of vortices ordered in a hexagonal lattice (snapshots 4, 5 and 6) or stripes (snapshots 7, 8, and 9) persists up to larger currents. With a further current increase, the vortex stripes hinder the development of vortex rivers such that an ordered vortex-stripe state is preserved up to yet larger transport currents (snapshots 8 and 9) as compared to the case of a hexagonal vortex lattice. A hallmark of the stripe symmetry is that the vortex lattice maintains its stripe-like ordering in a broad range of transport currents despite the same amplitude of the eddy currents.

On the basis of this discussion, we propose the following mechanism of coherent magnon–fluxon coupling: A moving vortex lattice in the superconductor exposes the adjacent ferromagnetic layer to a periodically modulated magnetic stray field, varying both in time and space. This field interacts with the ferromagnet through two primary mechanisms: (1) the induction of eddy currents and (2) the excitation of spin precession. In a dirty Fe–Co ferromagnet with high resistivity, the induced eddy currents are weak and rapidly decay, making magnon excitation the dominant interaction mechanism. Conversely, the excited magnons in Co–Fe generate dynamic magnetic fields, which act back on the superconductor by inducing eddy currents. These currents become locked to the moving vortex lattice, resulting in the appearance of a Shapiro step. This effect of eddy currents is consistent with the observed enhancement of the vortex instability velocity ${v}_{{\rm{m}}}^{* }$, which exceeds the instability velocity in the reference state, $v^{*}$, by about 10% (Fig. 3e). The numerical simulations of the vortex-lattice structure corroborate an enhanced vortex lattice ordering in the regime of magnon generation (Supplementary Note 2 and Extended Data Fig. 1).

## Micromagnetic simulations of magnon generation

**Fig. 4:**
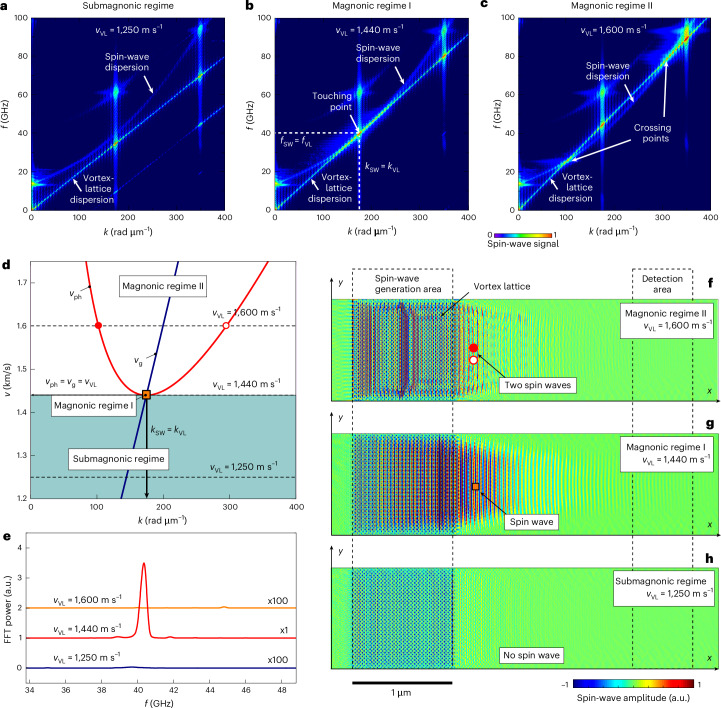
Micromagnetic simulations of the magnon generation by a moving fluxon lattice. **a**–**c**, Dispersion curves for magnons at *H*_ext_ = 1.85 T and the vortex lattice moving with different velocities in the submagnonic regime (**a**), magnonic regime I (**b**) and magnonic regime II (**c**). **d**, Dependence of the phase (*v*_ph_) and group (*v*_g_) velocities of magnons on the wavenumber *k*. In the submagnonic regime (shaded region), there is no magnon generation. Magnonic regime I corresponds to a monochromatic and unidirectional magnon excitation at the frequency and momentum matching condition, *v*_VL_ = *v*_SW_ = *v*_ph_ = *v*_g_ and *k*_VL_ = *k*_SW_, respectively. In magnonic regime II, two spin waves are generated, moving in the same direction as the vortex lattice, one in front of it and one behind it. **e**, Magnon frequency spectra in the detection area for the three vortex lattice velocity values. Multiplication factors are indicated next to the spectra. **f**–**h**, Generation and propagation of spin waves in the Co–Fe conduit in the magnonic regime II (**f**) and magnonic regime I (**g**) in comparison with the submagnonic regime (**h**) where no spin wave is generated.

To analyse the excitation of magnons by the moving vortex lattice, we performed micromagnetic simulations using the MuMax3 solver^41^ (Supplementary Note 1). In our geometry, the magnon dispersion relation can be approximated by the quadratic law $${f}_{{\rm{SW}}}({k}_{{\rm{SW}}})={f}_{{\rm{FMR}}}$$$$+{\tilde{f}}_{{\rm{SW}}}({k}_{{\rm{SW}}})$$ with $${\tilde{f}}_{{\rm{SW}}}({k}_{{\rm{SW}}})\backsim A{k}_{{\rm{SW}}}^{2}$$ (Fig. 4a). Here, the ferromagnetic resonance frequency *f*_FMR_ determines the minimal frequency at *k*_SW_ = 0, and *A* is the exchange constant. The dispersion for the moving vortex lattice is linear, with $$f_{\rm{VL}}=v_{\rm{VL}}/a_{\rm{VL}}=v_{\rm{VL}}k_{\rm{VL}}/2{\uppi}$$ (ref. ^16^). Accordingly, an increase of *v*_VL_ leads to a steeper slope of *f*_VL_(*k*_VL_), which eventually intersects the parabola (Fig. 4b). The condition for the dispersion crossing is *f*_SW_ = *f*_VL_ and *k*_SW_ = *k*_VL_. The touch/cross point of the two dispersions represents the magnon generation condition. With a further increase of *v*_VL_, *f*_VL_(*k*_VL_) intersects the parabola at two points (Fig. 4c). A closer insight into the physics of magnon generation by a lattice of fluxons can be gained from a consideration of the dependencies *v*_SW_(*k*_SW_) deduced from the dispersion curves.

Figure 4d shows the dependence of the magnon phase and group velocities, $v_{\rm{ph}}=2{\pi}f_{\rm{SW}}/k_{\rm{SW}}$ and $v_{\rm{g}}=2{\pi}{\partial}f_{\rm{SW}}/{\partial}k_{\rm{SW}}$, on the wavenumber *k*_SW_. In this representation, different vortex lattice velocities correspond to the crossings of *v*_ph_(*k*_SW_) and *v*_g_(*k*_SW_) at different *v* levels parallel to the *k* axis. At the crossing/touch point, which we call magnonic regime I, not only is *v*_VL_ equal to *v*_g_ but also *v*_g_ = *v*_ph_. These equalities mean that the energy from the vortex lattice is spent for a monochromatic excitation of magnons, which, in addition, are excited unidirectionally, that is, propagate only in one direction, which is determined by the direction of motion of the vortex lattice. This behaviour explains the observed unidirectionality of the magnon generation. Previously, unidirectional excitation of spin waves was achieved using nanoscale magnetic gratings^42^, surface acoustic waves^43^ and circularly polarized light pulses^44^. In our studies, the unidirectionality and monochromaticity are achieved through the momentum and energy matching conditions, *k*_SW_ = *k*_VL_ and *f*_SW_ = *f*_VL_.

When *v*_VL_ exceeds the threshold velocity of the magnon generation, two magnons are excited with different group velocities and wavelengths (Fig. 4f). In this regime, which we call magnonic regime II, one magnon moves faster than the vortex lattice and the other moves slower. However, owing to these wavelengths’ very weak excitation and detection efficiency, their intensities are negligibly small (Fig. 4e). By contrast, we reveal a pronounced single-wavelength magnon generation in magnonic regime I (Fig. 4g), and no magnon generation in the submagnonic regime (Fig. 4h). For the respective animations, the reader is referred to Supplementary Videos 1–3.

Finally, in Extended Data Fig. 1 we show the evolution of vortex lattice configurations upon generation of magnons (sub 40 nm), using the modified time-dependent Ginzburg–Landau (TDGL) equation in conjunction with the heat-balance equation for our simulations (Supplementary Note 2). Without a magnonic conduit, as the current increases, areas with a suppressed order parameter nucleate at the Nb–C edge where the vortices enter the superconductor. When magnon excitation is modelled with a resonance-like enhancement of eddy currents, the *I*–*V* curve becomes flattened, resembling a Shapiro step, and the ordered motion of vortices continues up to higher currents. Therefore, in the TDGL simulations, we have reproduced the effect of suppressing flux-flow instability in the regime of magnon generation.

## Conclusions

We demonstrate that a moving lattice of Abrikosov vortices in a superconductor excites spin waves in an adjacent ferromagnetic layer. The resulting magnon generation is both monochromatic and unidirectional, enabled by energy and momentum matching between fluxons and magnons. We identify a magnon Shapiro step in the current–voltage characteristics of the superconductor as a hallmark of self-locked, coherent magnon–fluxon dynamics. Moreover, magnon generation preserves the long-range order of the vortex lattice and enhances the superconductor’s current-carrying capability. In our experiments, we utilize Co–Fe as a proof-of-principle magnonic system to demonstrate this mechanism of magnon excitation. For future studies, other materials with longer magnon decay lengths, such as yttrium iron garnet, may be considered. In addition to microwave detection, observing the emitted magnons using low-temperature Brillouin light scattering (BLS) imaging would be of interest. However, aside from the technical challenges of conducting BLS measurements at cryogenic temperatures, a fundamental limitation remains: the diffraction-limited spatial resolution of BLS is approximately 250 nm, notably larger than the 35–40 nm wavelengths of the spin waves observed in our system, rendering them unresolvable by this technique. Overall, our findings enhance the understanding of magnon generation beyond conventional inductive methods and provide a basis for integration into advanced electronic and hybrid quantum systems. The demonstrated mechanism also exemplifies the broader interplay between superconductivity and magnetism, highlighting the potential of coupled dynamic excitations in engineered nanoscale platforms.

## Methods

### Fabrication of the microwave nano-antenna

The fabrication of the experimental system began with the deposition of a 40/5 nm Au/Cr film onto a Si (100 nm, p-doped)/SiO_2_ (200 nm) substrate and its patterning for electrical d.c. current and microwave measurements. In the sputtering process, the substrate temperature was 22 °C, the growth rates were 0.055 nm s^−1^ and 0.25 nm s^−1^, and the Ar pressures were 2 × 10^−3^ mbar and 7 × 10^−3^ mbar for the Cr and Au layers, respectively. The microwave ladder antenna was fabricated from the Au/Cr film by focused ion beam milling at 30 kV/30 pA in a dual-beam scanning electron microscope (FEI Nova Nanolab 600). The multi-element antenna consisted of ten nanowires connected in parallel between the signal and ground leads of a 50-Ω-matched microwave transmission line. The antenna had a period *p* = 108 nm with the nanowire width equal to the nanowire spacing so that its Fourier transform contained only odd spatial harmonics with${\lambda}_{1}=p$ and ${\lambda}_{3}=p/3=36\,{\rm{nm}}$, which made it sensitive to spin-wave wavelengths of 36 ± 2 nm in our experiments (Supplementary Fig. 1).

### Fabrication and properties of the Nb–C microstrip

The ladder antenna’s fabrication was followed by direct writing of the superconducting strip at 2 μm (edge to edge) from the microwave antenna. The 45-nm-thick Nb–C microstrip was fabricated by focused ion beam-induced deposition. Focused ion beam-induced deposition was done at 30 kV/10 pA, 30 nm pitch and 200 ns dwell time employing Nb(NMe_2_)_3_(N-*t*-Bu) as precursor gas^26^. The superconducting strip and the ladder antenna were covered with a 3-nm-thick insulating Nb–C layer prepared by focused electron beam-induced deposition (FEBID). Before the Co–Fe magnonic waveguide deposition, a 48-nm-thick insulating Nb–C–FEBID layer was deposited to compensate for the structure height variations between the antenna and the Nb–C strip. The elemental composition in the Nb–C strip was 45 ± 2 at.% C, 29 ± 2 at.% Nb, 15 ± 2 at.% Ga and 13 ± 2 at.% N, as inferred from energy-dispersive X-ray spectroscopy on thicker microstrips written with the same deposition parameters. The Nb–C strip had well-defined smooth edges and a root mean squared surface roughness of <0.3 nm, as deduced from atomic force microscopy scans over its 1 μm × 1 μm active part before the deposition of the Co–Fe layer. The two ends of the strip had rounded sections to prevent current crowding effects at the sharp strip edges, which may lead to an undesirable reduction of the experimentally measured critical current and the instability current^45^.

The resistivity of the Nb–C microstrip at 7 K was $\rho_{7{\rm{K}}}=551\,\upmu{\Omega}\,{\rm{cm}}$. The microstrip transitioned to a superconducting state below the transition temperature *T*_c_ = 5.60 K, deduced using a 50% resistance drop criterion. Application of a magnetic field *H*_ext_ ≈ 2 T led to a decrease of *T*_c_(0) to *T*_c_(2 T) ≈ 5.1 K. Near *T*_c_, the critical field slope $(\mathrm{d}{H}_{{\rm{c2}}}/\mathrm{d}T){| }_{{T}_{{\rm{c}}}}=-2.19$ T K^−1^ corresponds, in the dirty superconductor, to the electron diffusivity $D=-4{k}_\mathrm{B}/(\pi e(\mathrm{d}{H}_{{\rm{c2}}}/\mathrm{d}T){| }_{{T}_{{\rm{c}}}})\approx 0.5$ cm^2^ s^−1^ with the extrapolated zero-temperature upper critical field value *H*_*c*2_(0) ≈ 12.3 T. Here, *k*_B_ is the Boltzmann constant and *e* the elementary charge. The coherence length and the penetration depth at zero temperature were estimated^46^ as ${\xi }_{\mathrm{c}}={(\hslash D/{k}_{\mathrm{B}}{T}_{\mathrm{c}})}^{1/2}\approx 9\,{\mathrm{nm}}$ (corresponding to ${\xi}(0)={\xi}_{\rm{c}}{(1.76)}^{-1/2}\approx7\,{\rm{nm}}$) and $\lambda (0)=1.05\times 1{0}^{-3}{({\rho }_{{\rm{7K}}}{k}_\mathrm{B}/\varDelta (0))}^{1/2}\approx \text{1,040}\,{\mathrm{nm}}$. Here, ${\varDelta}(0)$ is the zero-temperature superconducting energy gap and $\hslash$ is the Planck constant.

### Fabrication and properties of the Co–Fe conduit

The Co–Fe magnonic conduit was 1μm wide, 5 μm long and 30 nm thick. We fabricated it by FEBID employing HCo_3_Fe(CO)_12_ as the precursor gas^47^. FEBID was done with 5 kV/1.6 nA, 20 nm pitch and 1 μs dwell time. The material composition in the magnonic waveguide is 61 ± 3 at.% Co, 20 ± 3 at.% Fe, 11 ± 3 at.% C and 8 ± 3 at.% C. The oxygen and carbon are residues from the precursor in the FEBID process^47^. The Co–Fe conduit consisted of a dominating bcc Co_3_Fe phase mixed with a minor amount of FeCo_2_O_4_ spinel oxide phase with nanograins of about 5 nm in diameter. The random orientation of Co–Fe grains in the carbonaceous matrix ensured negligible magnetocrystalline anisotropy. Further details on the microstructural and magneto-transport properties of Co–Fe–FEBID were reported previously^47^.

### Electrical resistance measurements

The *I*–*V* curves were recorded in current-driven mode within a ^4^He cryostat fitted with a superconducting solenoid. The external magnetic field *H*_ext_ was tilted at a small angle $\beta={5}^{\circ}$ with respect to the normal to the sample plane (*z* axis) in the plane perpendicular to the direction of the transport current. The small field tilt angle $\beta$ ensures that the field component *H*_ext,*z*_ acting along the *z* axis is only negligibly smaller than *H*_ext_ with (*H*_ext_ − *H*_ext,*z*_)/*H*_ext_ ≤ 0.5%. The transport current applied along the *y* axis in a magnetic field *H* ≈ *H*_ext_ = *H*_*z*_ exerts on vortices a Lorentz force acting along the *x* axis^28^. The voltage induced by the vortex motion across the superconducting microstrip was measured with a nanovoltmeter. A series of reference measurements was taken before the deposition of the Co–Fe magnonic conduit on top of the Nb–C strip. No voltage steps were revealed in the *I*–*V* curves of the bare Nb–C strip. By contrast, constant-voltage steps in the *I*–*V* curves were revealed after the deposition of the Co–Fe magnonic conduit on top of the Nb–C strip. The rarely achieved combination of weak volume pinning, in conjunction with close-to-perfect edge barriers and a fast relaxation of nonequilibrium electrons, allows for ultrafast motion of Abrikosov vortices in the Nb–C superconductor^31^.

### Microwave detection of spin waves

The microwave detection of spin waves was performed using a microwave ladder nano-antenna connected to a spectrometer system. This allowed detecting signals at power levels down to 10^−16^ W in a 25 MHz bandwidth^32^. The detector system consisted of a spectrum analyser (Keysight Technologies N9020B, 10–50 GHz), a semirigid coaxial cable (SS304/BeCu, d.c.–61 GHz) and an ultrawide-band low-noise amplifier (RF-Lambda RLNA00M54GA, 0.05–54 GHz, +20 dB gain).

### Micromagnetic simulations

The micromagnetic simulations were performed using the graphics processing unit-accelerated simulation package MuMax3 to calculate the investigated structures’ space- and time-dependent magnetization dynamics^41^. The simulations were done for the following Co–Fe parameters: saturation magnetization *M*_s_ = 1.4–1.5 MA m^−1^, exchange constant *A* = 15–18 pJ m^−1^ and Gilbert damping ${\alpha}=0.01$. The best match of the simulation results with the experimental data has been revealed for *M*_s_ = 1.45 MA m^−1^ and *A* = 15 pJ m^−1^. The mesh was set to 2 × 2 nm^2^, which is smaller than the exchange length of Co–Fe (~5 nm) and fulfils the requirements for micromagnetic simulations. The simulations were validated by comparing with the results obtained within the Kalinikos–Slavin theory^48^ (Supplementary Fig. 3). The estimated attenuation length of the generated magnons (at *k*_SW_ ≈ 175 rad μm^−1^) is around 600 nm (ref. ^48^).

An external field *H*_ext_ in the range 1.75–1.95 T, sufficient to magnetize the structure to saturation, was applied at a small angle $\beta$ relative to the *z* axis in the *x–z* plane (Supplementary Fig. 4). A fast-moving periodic field modulation was used to mimic the effect of the moving vortex lattice. The oscillations *m*_*x*_(*x*, *y*, *t*) were calculated for all cells and all times via *m*_*x*_(*x*, *y*, *t*) = *M*_*x*_(*x*, *y*, *t*) − *M*_*x*_(*x*, *y*, 0), where *M*_*x*_(*x*, *y*, 0) corresponds to the ground state (fully relaxed state without any moving magnetic field source). The dispersion curves were obtained by performing two-dimensional fast Fourier transformations of the time- and space-dependent data. The spin-wave spectra were calculated by performing a fast Fourier transformation of the data in a region at a distance of 1 μm from the spin-wave excitation region (Supplementary Fig. 5). Full details on the micromagnetic simulations are given in Supplementary Note 1. The evolution of the magnon generation condition upon variation of the magnetization, exchange stiffness and thickness of the Co–Fe conduit is illustrated in Supplementary Figs. 6–9. The observed magnon generation may be interpreted by two scenarios, namely the coherent fluxon-magnon coupling and the magnonic Cherenkov effect. The relation between the Cherenkov effect for single and multiple periodically arranged moving particles is illustrated in Supplementary Fig. 10.

### Ginzburg–Landau simulations

The evolution of the superconducting order parameter ${\varDelta}=|{\varDelta}|{e}^{i{\varPhi}}$ was analysed relying upon a numerical solution of the modified TDGL equation^49^, solved in conjunction with the heat-balance equation, to account for possible heating effects

$$\begin{array}{r}\displaystyle\frac{\pi \hslash }{8{k}_{{\rm{B}}}{T}_{{\rm{c}}}}\left(\displaystyle\frac{\partial }{\partial t}+\frac{2ie\varphi }{\hslash }\right)\varDelta =\\ ={\xi }_{{\rm{mod}}}^{2}{\left(\nabla -i\displaystyle\frac{2e}{\hslash c}\mathbf{A}\right)}^{2}\varDelta +\left(1-\displaystyle\frac{{T}_{{\rm{e}}}}{{T}_{{\rm{c}}}}-\displaystyle\frac{| \varDelta {| }^{2}}{{\Delta }_\mathrm{mod}^{2}}\right)\varDelta +\\ +i\displaystyle\frac{({\rm{div}}\,{{\bf{j}}}_{{\rm{s}}}^{{\rm{Us}}}-{\rm{div}}\,{{\bf{j}}}_{{\rm{s}}}^{{\rm{GL}}})}{| \varDelta {| }^{2}}\frac{e\varDelta \hslash D}{{\sigma }_{{\rm{n}}}\sqrt{2}\sqrt{1+{T}_{{\rm{e}}}/{T}_{{\rm{c}}}}},\end{array}$$

where $${\xi }_{{\rm{mod}}}^{2}=\pi \sqrt{2}\hslash D/(8{k}_{{\rm{B}}}{T}_{{\rm{c}}}\sqrt{1+{T}_{{\rm{e}}}/{T}_{{\rm{c}}}})$$, $${\varDelta }_{{\rm{mod}}}^{2}={({\varDelta }_{0}\tanh (1.74\sqrt{{T}_{{\rm{c}}}/{T}_{{\rm{e}}}-1}))}^{2}$$$$/(1-{T}_{{\rm{e}}}/{T}_{{\rm{c}}})$$, **A** is the vector potential, *φ* is the electrostatic potential, *D* is the diffusion coefficient, $${\sigma}_{\rm{n}}=2{e}^{2}DN(0)$$ is the normal-state conductivity with *N*(0) being the single-spin density of states at the Fermi level, *T*_e_ and *T*_p_ are the electron and phonon temperatures, and $${{\bf{j}}}_{{\rm{s}}}^{{\rm{Us}}}$$ and $${{\bf{j}}}_{s}^{{\rm{GL}}}$$ are the superconducting current densities in the Usadel and Ginzburg–Landau models

1 $${{\bf{j}}}_{{\rm{s}}}^{{\rm{Us}}}=\frac{\pi {\sigma }_{{\rm{n}}}}{2e\hslash }| \varDelta | \tanh \left(\frac{| \varDelta | }{2{k}_{{\rm{B}}}{T}_{{\rm{e}}}}\right){{\bf{q}}}_{{\rm{s}}},$$

where $${\bf{q}}_{\rm{s}}={\nabla}{\varphi}+2{\pi}{\bf{A}}/{\varPhi}_{0}$$ and $${{\bf{j}}}_{{\rm{s}}}^{{\rm{GL}}}=\frac{\pi {\sigma }_{{\rm{n}}}| \varDelta {| }^{2}}{4e{k}_{{\rm{B}}}{T}_{{\rm{c}}}\hslash }{{\bf{q}}}_{{\rm{s}}}$$.

The vector potential **A** = (0, *A*_y_, 0) in the TDGL equation consists of two parts: *A*_y_ = *H*_ext_*x* + *A*_m_, where *H*_ext_ is the external magnetic field and *A*_m_ is the vector potential of the magnetic field induced in the superconducting strip by spin waves.

The modelled length of the microstrip is *L* = 4*w*, the width $w=50{\xi}_{c}$, and the parameter ${B}_{0}={\varPhi}_{0}/(2{{\pi}}{\xi}_{\rm{c}}^{2})\approx 4.15\,{\rm{T}}$, where ${\xi}_{c}=8.9\,{\rm{nm}}$. The calculations were done with parameters $\gamma=9$ and ${\tau}_{0}=925\,{\rm{ns}}$ for NbN, as their values for NbC are unknown but are supposed to be of the same order of magnitude. A variation of $\gamma$ and $\tau$ only leads to quantitative changes in the *I*–*V* curves and does not qualitatively change the vortex dynamics. In simulations, *dA* was varied between 0 (no ferromagnet layer) and $0.1{\varPhi}_{0}/(2{\pi}{\xi}_{\rm{c}})$, which corresponds to about 1/4 of the depairing velocity for superconducting charge carriers (Cooper pairs) or critical $q_{\rm{sc}}0.35\,{\varPhi}_{0}/2{\pi}{\xi}_{\rm{c}}$ of the superconducting strip at *B* = 0 and *T* = 0.8*T*_c_. The parameters *a*_*x*_ and *a*_*y*_ were chosen to model a triangular moving vortex lattice without the ferromagnetic layer and far from the instability point. We present the results for ${v}=110{\xi}_{\rm{c}}/{\tau}_{0},\,{a}_{x}=5.5{\xi}_{\rm{c}}$ and ${a}_{y}=9.2{\xi}_{\rm{c}}(a_{\rm{VL}}=4.9{\xi}_{\rm{c}}\,{\rm{at}}\, B=0.3\,B_{0})$. We find that the width and the slope of the plateau in the *I*–*V* curve weakly vary with small variations of *a*_*x*_ and *a*_*y*_, while the value of *v*_m_ controls the voltage plateau position.

Further details on the TDGL simulations are given in Supplementary Note 2. The TDGL modelling results are presented in Extended Data Fig. 1. The animated spatiotemporal evolutions of the superconducting order parameter are shown in Supplementary Videos 4–12.

## Online content

Any methods, additional references, Nature Portfolio reporting summaries, source data, extended data, supplementary information, acknowledgements, peer review information; details of author contributions and competing interests; and statements of data and code availability are available at 10.1038/s41565-025-02024-w.

## Supplementary information

Supplementary Information Supplementary Notes 1–3, Figs. 1–10, captions to Videos 1–12 and References.

Supplementary Video 1 Spatiotemporal evolution of the magnetization component *m*_x_ in the Co–Fe magnonic waveguide upon as the vortex lattice moves with velocity *v*_V L_ = 1,250 m s^−1^ in the Nb–C superconductor (submagnonic regime).

Supplementary Video 2 Spatiotemporal evolution of the magnetization component *m*_x_ in the Co–Fe magnonic waveguide upon as the vortex lattice moves with velocity *v*_V L_ = 1,440 m s^−1^ in the Nb–C superconductor (magnonic regime I).

Supplementary Video 3 Spatiotemporal evolution of the magnetization component *m*_x_ in the Co–Fe magnonic waveguide upon as the vortex lattice moves with velocity *v*_V L_ = 1,600 m s^−1^ in the Nb–C superconductor (magnonic regime II).

Supplementary Video 4 Spatiotemporal evolution of the superconducting order parameter in the bare Nb-C superconducting strip at the transport current *I* = 0.11*I*_dep_ (depairing current of the superconductor).

Supplementary Video 5 Spatiotemporal evolution of the superconducting order parameter in the bare Nb-C superconducting strip at the transport current *I* = 0.12*I*_dep_.

Supplementary Video 6 Spatiotemporal evolution of the superconducting order parameter in the bare Nb-C superconducting strip at the transport current *I* = 0.13*I*_dep_.

Supplementary Video 7 Spatiotemporal evolution of the superconducting order parameter for the assumed hexagonal lattice of vortices in the Nb–C superconducting strip overlaid by the Co–Fe magnonic conduit at the transport current *I* = 0.11*I*_dep_.

Supplementary Video 8 Spatiotemporal evolution of the superconducting order parameter for the assumed hexagonal lattice of vortices in the Nb–C superconducting strip overlaid by the Co–Fe magnonic conduit at the transport current *I* = 0.12*I*_dep_.

Supplementary Video 9 Spatiotemporal evolution of the superconducting order parameter for the assumed hexagonal lattice of vortices in the Nb–C superconducting strip overlaid by the Co–Fe magnonic conduit at the transport current *I* = 0.13*I*_dep_.

Supplementary Video 10 Spatiotemporal evolution of the superconducting order parameter for the assumed stripe-like periodic arrangement of vortices in the Nb–C superconducting strip overlaid by the Co–Fe magnonic conduit at the transport current *I* = 0.11*I*_dep_.

Supplementary Video 11 Spatiotemporal evolution of the superconducting order parameter for the assumed stripe-like periodic arrangement of vortices in the Nb–C superconducting strip overlaid by the Co–Fe magnonic conduit at the transport current *I* = 0.12*I*_dep_.

Supplementary Video 12 Spatiotemporal evolution of the superconducting order parameter for the assumed stripe-like periodic arrangement of vortices in the Nb–C superconducting strip overlaid by the Co–Fe magnonic conduit at the transport current *I* = 0.13*I*_dep_.

## Data Availability

The data supporting the findings of this study are available within the article and Supplementary Information. These data are also available via Mendeley Data at https://data.mendeley.com/datasets/7hy5n477tj/1 (ref. ^50^).
