# Supplementary material for: Moving Abrikosov vortex lattices generate sub-40-nm magnons
Source: Nat Nanotechnol. 2025 Oct 16;20(12):1764–70. doi: 10.1038/s41565-025-02024-w (PMC12727539; doi:10.1038/s41565-025-02024-w)
Supplement: Supplementary file 1 — Supplementary Notes 1–3, Figs. 1–10, captions to Videos 1–12 and References. [file 41565_2025_2024_MOESM1_ESM.pdf]

nature nanotechnology

Supplementary information https://doi.org/10.1038/s41565-025-02024-w

Moving Abrikosov vortex lattices generate sub-40-nm magnons

In the format provided by the authors and unedited

Supplementary Notes

Supplementary Note 1: Micromagnetic simulations

The micromagnetic simulation results were first validated by comparing them with the results of analytical calculations. Specifically, the spin-wave dispersion curve for the Co-Fe conduit was compared with the dispersion curve calculated within the Kalinikos-Slavin theory [1]. It should be noted that the relevant angle in the Kalinikos-Slavin theory is the angle θ at which the effective magnetic field H_{eff} is tilted relative to the z-axis. This angle θ and the effective field H_{eff} were extracted from micromagnetic simulations, using boundary conditions of fully pinned spins at the edges of the Co-Fe conduit. The dispersion curves calculated using the analytical Kalinikos-Slavin theory fit the simulation results very well, as illustrated in Supplementary Fig. 3.

In general, a moving modulation landscape of the out-of-plane magnetic field characterises a moving vortex lattice in the superconductor. This periodically time- and space-modulated magnetic field interacts with adjacent materials through two mechanisms: (i) the induction of eddy currents and (ii) the excitation of spin precessions. In Fe-Co, a dirty ferromagnet created by FEBID has a large resistance, and eddy currents decay fast. Thus, the excitation of magnons is the dominant mechanism at sufficiently high vortex velocities (equal to or exceeding the magnon phase velocity). Conversely, the excited magnons in Co-Fe are characterised by the dynamic components of the magnetic field, which act back on the superconductor by inducing eddy currents that are, in turn, locked to the moving vortex lattice, thus giving rise to the Shapiro step. More details on the magnon Shapiro steps are given in Supplementary Note 2.

Specifically, to model the generation of magnons by a moving vortex lattice inducing a spatially modulated out-of-plane magnetic field $h_z(x,y)$, we apply an out-of-plane field $h_z(x,y)$ with a square-shaped profile (10 mT amplitude, 2×2 nm² area per unit) arranged in a hexagonal lattice with a 36 nm periodicity. Next, we move $h_z(x,y)$ at the same velocity as the vortex lattice and record the x-component of the magnetisation $m_x(x,y,t)$ across all cells in the magnetic layer. To analyse the dynamics, we perform a two-dimensional Fourier transform along the propagation direction (x) and time, then average over the y-direction to obtain the dispersion relation $m_x(k_x,f)$, as shown in Fig. 4a of the main text. Additionally, we plot spatial snapshots $m_x(x,y,t_i)$ at specific

times t_i, illustrated in Fig. 4f of the main text. In short, the moving out-of-plane magnetic field induced by the moving vortex lattice is introduced into the magnetic layer for the excitation of magnons.

Regarding the influence of the out-of-plane field component induced by vortices, our analysis shows that while this component can excite magnons across a broad frequency range, its amplitude is a few orders of magnitude smaller than the time-dependent magnetisation perturbations induced by the vortex motion, thus the direct contribution of the *z*-component of the vortex field is negligible. In the investigated range of fields of about 2 T, the vortex lattice is dense, and the modulation of the local magnetic field along the *z*-component at the vortex cores and between them is small, with $\Delta B_z \approx 0.1$ mT. The magnetic penetration depth λ (~1 μm) is much larger than the Nb-C strip's vortex-lattice parameter a_{VL} (≈35 nm). However, the other components of the field modulation also contribute to the spin-wave excitation. Given a large number of vortices (850-950, depending on the applied magnetic field value) threading the 1 μm x 1 μm part of the superconductor underneath the Co-Fe magnonic conduit at the magnetic fields of interest, such a small modulation of the magnetic field induced by the moving vortex lattice was enough to excite spin waves propagating over the 2 μm distance between the superconducting microstrip and the microwave antenna. Within the framework of the Kalinikos-Slavin theory [1], the spin-wave decay length was estimated as 600 nm at the wavenumber k_{SW} = 175 rad/μm.

In the experiment, the external field H_{ext} was directed at a small tilt angle β with respect to the normal to the sample plane. The angle β lay in the *xz*-plane and was nominally set to $\beta = 5°$ in the experiment. It should be noted that this geometry is distinct from the limiting case of forward volume spin-wave (FVSW) geometry with $\beta = 0°$, see Supplementary Fig. S4. Thus, we call this configuration quasi-FVSW. The magnetisation **M** of the Co-Fe conduit at $\beta \neq 0°$ is directed not along $\mathbf{H}_{\mathrm{ext}}$ but along the effective field $\mathbf{H}_{\mathrm{eff}}$ tilted at the angle θ away from the *z*-axis. Namely, for $\beta = 5°$, the angle θ is about 30°. Note, the angle θ and the effective field H_{eff} depend strongly on the angle β, see Supplementary Fig. 6(a). The dependences $\theta(\beta)$ were deduced from the micromagnetic simulations for a series of values of the saturation magnetisation M_{s}, exchange stiffness A, and the thickness of the Co-Fe waveguide.

The magnon generation condition implies a match between the energy and momentum, which is satisfied at the point of intersection of the two dispersion curves (straight line for the vortex lattice and parabola for the spin waves). To facilitate the experimental observation of this resonance, a magnetic field tilt angle β~5° was essential to slightly “flatten” (or, in other words, “linearise”) the parabolic dispersion for spin waves, to expand the fluxon-magnon interaction over some range of wavevectors and vortex velocities, to mitigate possible effects of structure imperfections and field misalignment. We note that the angle θ of the effective field relative to the normal to the sample plane depends strongly on β at β~0, and the dependence $\theta(\beta)$ is already weak enough for β~4-5°, see panels (d) and (e) in Supplementary Fig. 4.

Various spatial field profiles and arrangements of vortices, induced by moving magnetic perturbations, were used in the simulations. Specifically, the excitation of spin waves was investigated for sawtooth, cosine, and meander-like magnetic induction profiles, as well as vortices arranged in hexagonal, square, and squeezed-square (stripe-like pattern) lattices. A field modulation induced by a moving array of periodically arranged vortex stripes achieved the largest spin-wave amplitude. In contrast, the smallest spin-wave amplitude was obtained for a hexagonal vortex lattice, Supplementary Fig. 5.

By fixing the thickness t of the Co-Fe magnonic conduit to 30 nm, as deduced from atomic force microscopy measurements, and considering the external field H_{ext} = 1.85 T as an exemplary field value at which spin waves with k = 175 rad/µm have been detected, the variation of M_s and A as two fitting parameters allowed for matching (horizontal and vertical lines between the panels in Supplementary Fig. 6) of the spin-wave frequency and velocity at the magnon generation condition for β = 4.7°. This deduced value of β is only by 0.3° smaller than the field tilt angle in the experiment, and it corresponds to the angle θ = 29° (see panel (a) in Supplementary Fig. 6) between the direction of the magnetisation M and the z-axis. The best-fit parameters M_s = 1450 kA/m and A = 17 pJ/m are used in the manuscript for quantitatively describing the magnon generation by the fast-moving lattice of magnetic flux quanta. The dependence of the magnon generation condition on the magnetisation, exchange stiffness and thickness of the Co-Fe conduit is illustrated in Supplementary Figs. 7-9.

The propagation of spin waves excited by the moving vortex lattice in the three regimes discussed in the main manuscript text is further illustrated in videos 1 to 3.

Overall, our experimental findings may be interpreted by two scenarios, namely a) coherent coupling between fluxons in the superconducting stripe with short-wavelength magnons in the magnetic conduit and b) magnonic Cherenkov effect in a hybrid system consisting of superconductor and ferromagnet separated by an insulating layer. The relation between the Cherenkov effect for single and multiple periodically arranged moving particles is illustrated in Supplementary Fig. 10, describing the Cherenkov effect from a classical perspective and for a lattice of vortices (our experimental case).

In the experiment, the coupling strength between the fluxon and magnon subsystems (i.e. the amplitude and spatial distribution of the eddy currents) is a poorly accessible quantity. In the simulations, the introduction of the amplitude of the eddy currents as a free parameter allows us to demonstrate a continuous evolution of the *I*-*V* curves from the nonlinear conductivity regime II followed by the instability jump regime III (as for the bare superconductor reference sample, Fig. 1a in the main text) to the voltage step regime followed by the steep upturn regime II and the instability jump regime III in the *I*-*V* curve for the superconductor/ferromagnet heterostructure, see Extended Data Fig. 5. Additionally, the TDGL simulations illustrate that a higher instability current and instability velocity can be achieved upon the generation of magnons by fast-moving fluxons. The enhancement of and v_{m}^{*} occurs due to the transition of the moving vortex lattice to the vortex river regime is prevented by the eddy currents induced by the excited spin wave for both the vortex stripe and the vortex lattice symmetries.

Finally, regarding the sub-40 nm wavelengths (wavenumbers k_{SW}>170rad/μm) of the excited/detected spin waves, we should emphasise that the excitation of propagating spin waves with wavelengths below 100 nm represents a critical task of modern magnonics. Excitation of short-wavelength exchange spin waves is challenging because of low microwave-to-magnon conversion efficiencies at the sub-100 nm scale. Here, we have demonstrated a new paradigm for the excitation of short-wave (exchange) spin waves by Abrikosov vortices as fast-moving magnetic perturbations, with the shortest wavelengths achieved experimentally so far. In addition, due to the periodicity of the vortex lattice, the spin-wave excitation is unidirectional, with the spin-wave propagating in the direction of the vortex motion, and it is monochromatic, with the wavelength equal to the vortex lattice parameter. Thus, the magnon generation by fast-moving vortices is an excellent spin-wave source for magnonic applications at the nanoscale.

Supplementary Note 2: TDGL simulations

The evolution of the superconducting order parameter $\Delta = |\Delta|e^{i\phi}$ was analysed by relying upon a numerical solution of the modified TDGL equation [2], solved in conjunction with the heat-balance equation, to account for possible heating effects. To examine the role of the vortex-structure ordering along the *x* and *y* axes, or the *x*-axis only, two kinds of A_m were considered, respectively

$$A_m = dA_m \sin(2\pi(y-\upsilon_{Ch}t)/a_x)\sin(2\pi(y-\upsilon_{Ch}t)/a_y),$$

$$A_m = dA_m \sin(2\pi(y-\upsilon_{Ch}t)/a_x),$$

where υ_{Ch} is the threshold velocity of magnon generation and a_x and a_y are parameters of the order of a_{VL}. The first expression for A_m models the resonance response of the ferromagnet in the presence of a nearly triangular vortex lattice moving with the velocity υ_{Ch}. The second expression for A_m accounts for the assumed appearance of vortex stripes at the magnon generation condition. Physically, the second expression is connected to the much larger amplitude of spin waves (inducing a larger A_m) compared to spin waves excited by a triangular vortex lattice, as inferred from the micromagnetic simulations. The component of the vector potential A_m induces eddy currents in the superconductor, which affect the vortex motion. The amplitude dA_m controls the amplitude of the eddy currents in the considered model, as the relation between the superconducting eddy currents and A_m follows from the equation for j_s^{Us}.

The electron and phonon temperatures, T_e and T_p, were found from the solution of the following equations

$$\frac{\partial}{\partial t}\left(\frac{\pi^2 k_B^2 N(0) T_e^2}{3} - E_0 E_s(T_e, |\Delta|)\right) = \nabla k_s \nabla T_e - \frac{96\zeta(5) N(0) k_B^2}{\tau_0}\frac{T_e^5 - T_p^5}{T_c^3} + jE$$

$$\frac{\partial T_p^4}{\partial t} = -\frac{T_p^4 - T_e^4}{\tau_{esc}} + \gamma\frac{24\zeta(5)}{\tau_0}\frac{15}{\pi^4}\frac{T_e^5 - T_p^5}{T_c},$$

where $E_0 = 4N(0)(k_B T_c)^2$, $E_0 E_s(T_e, |\Delta|)$ is the change in the energy of electrons due to the transition to the superconducting state, k_s is the heat conductivity in the superconducting state

$$k_s = k_n\left(1 - \frac{6}{\pi^2 (k_B T_e)^3}\int_0^{|\Delta|}\frac{\varepsilon^2 e^{\varepsilon/k_B T_e} d\varepsilon}{(e^{\varepsilon/k_B T_e}+1)}\right),$$

$k_n = 2D\pi^2 k_B^2 N(0) T_e/3$ is the heat conductivity in the normal state, the term jE describes Joule dissipation, and τ_{esc} is the escape time of nonequilibrium phonons to the substrate. The parameter γ is defined as $\gamma = 8\pi^2 C_e(T_c)/(5C_p(T_c))$, where $C_e(T_c)$ and $C_p(T_c)$ are the heat capacities

of electrons and phonons at T=T_c, and the characteristic time τ_0 controls the strength of the electron-phonon and phonon-electron scattering.

The electrostatic potential φ was found from the current continuity equation

$$\mathrm{div}(j_s^{Us}+j_n)=0,$$

where $j_n = -\sigma_n \nabla\varphi$ is the normal current density. The boundary conditions at the microstrip edges, where vortices enter and exit it, were $j_n|_n = j_s|_n=0$ and $\partial Te/\partial n=0$, $\partial|\Delta|/\partial n=0$. At the edges along the current direction, the boundary conditions were T_e=T, $|\Delta|=0$, $j_s|_n=0$, $j_n|_n=I/wd$. The latter boundary conditions model the contact of the superconducting strip with a normal reservoir. This choice provides a way to inject the current into the superconducting microstrip in the modelling.

The animated spatiotemporal evolutions of the superconducting order parameter at points 1-9 in the *I*-*V* curve in Extended Data Fig. 1 are shown in Videos 4-12.

The simulated *I*-*V* curves for the bare superconducting strip and the superconductor/ferromagnet heterostructure are presented in Extended Data Fig. 1.

Supplementary Note 3: Microwave-stimulated superconductivity

Finally, the enhancement of *I** and *v** could also be possible because of microwave stimulation of superconductivity, see e.g. [4]. Microwave stimulation of superconductivity implies a nonequilibrium re-distribution of quasiparticles away from the edge of the superconducting gap in conjunction with a relatively long time of the quasiparticle relaxation τ_E. At frequencies of 30-40 GHz and without dc-driven vortices producing dissipation, this phenomenon is typically observed for elemental superconductors like In, Al, Sn, where τ_E is of the order of 10^{-8}-10^{-10} s (we refer, e.g., to page 208 in [5]). In our work, we use the dirty-limit superconductor Nb-C where τ_E is of the order of 10^{-11} s, which means that possible microwave-stimulation effects would require an order-of-magnitude higher frequencies, in the absence of dc-driven vortices. In Fig. 1 of [4] it was shown that in the presence of a dc current, the peak in the density of states is smeared so that it is very unlikely that any stimulation effect can be observed for Nb-C even at 100-200 GHz frequencies, especially at a high magnetic field of about 2 T inducing a very dense vortex lattice.

Supplementary Figures

(a) (b)

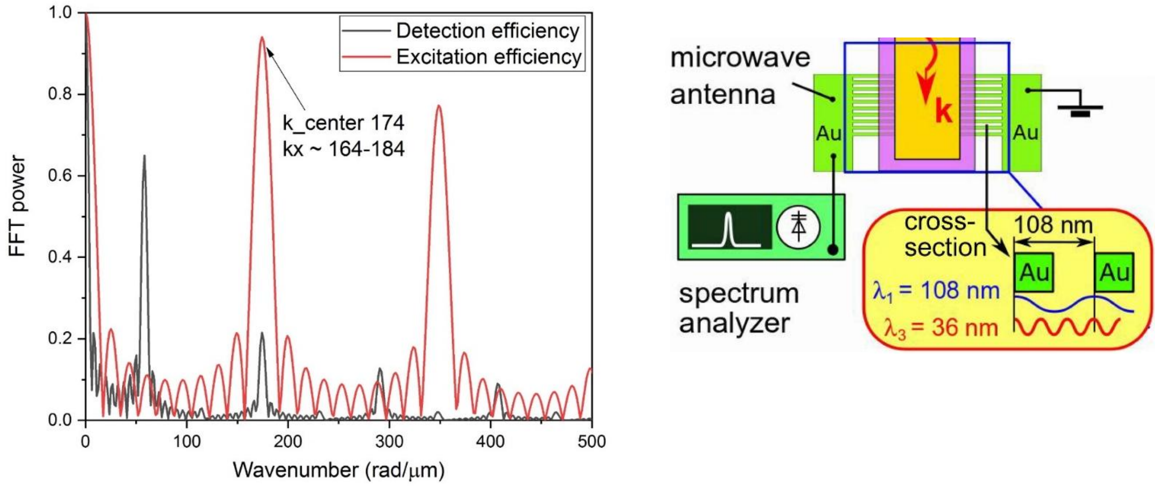

Supplementary Fig. 1. Spin-wave excitation and detection efficiency. (a) At H_{ext} = 1.85 T, the moving vortex lattice has peaks in the excitation efficiency at nk_{VL} = 175 rad/µm, where n is an integer. The detection antenna has a period p =108 nm, with the nanowire width equal to the nanowire spacing, so that its Fourier transform contains only odd spatial harmonics with $k_1 = 2\pi/p \approx 58$ rad/µm and $\lambda_3 = 6\pi/p \approx 174$ rad/µm. This makes the antenna sensitive to the spin-wave wavelengths of 36±2 nm in our experiments. (b) Spatial relations of the spin-wave wavelengths λ_1 and λ_3 with the period p =108 nm of the antenna.

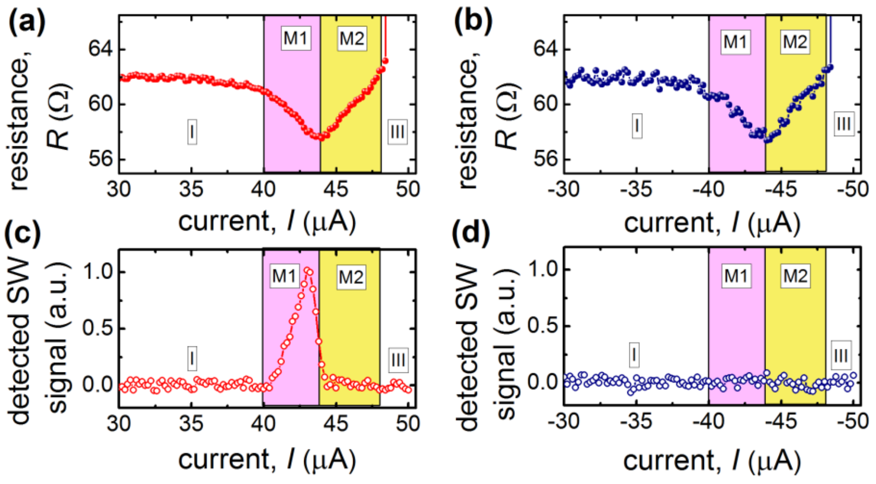

Supplementary Fig. 2. DC resistance and microwave detection upon current polarity reversal. Electrical resistance (panels (a) and (b)) and detected spin-wave signals (panels (c) and (d)) at 38.5 GHz for the positive (panels (a) and (c)) and negative (panels (b) and (d)) current polarity at H_{ext} = 1.81 T. The positive current polarity corresponds to the vortex motion toward the microwave nano-antenna. The negative current polarity corresponds to the vortex motion away from the nano-antenna. Note a resistance minimum at about -43.5 µA in panel (b) at which there is no microwave signal from the excited spin waves in panel (d).

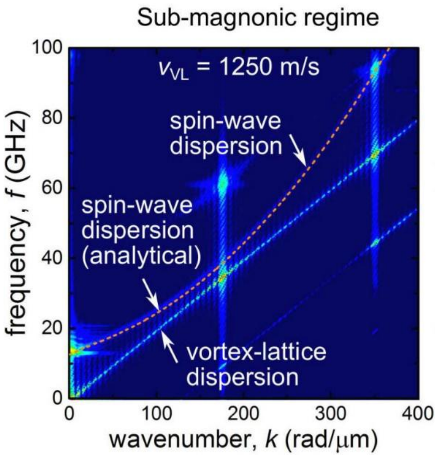

Supplementary Fig. 3. Dispersion curves for spin waves and the vortex lattice moving with velocity v_{VL} = 1250 m/s at H_{ext} = 1.85 T. The dashed magenta line is the spin-wave dispersion calculated within the Kalinikos-Slavin theory [1]. The vertical lines originate from the periodic structure of the vortex lattice and are unrelated to the magnon intensity.

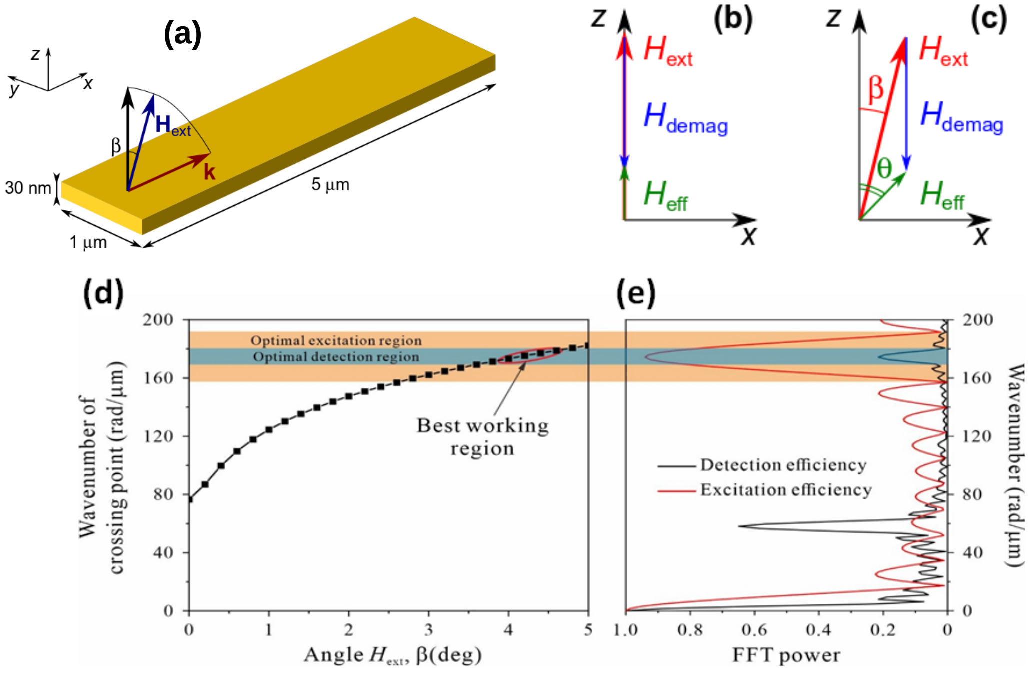

Supplementary Fig. 4. Geometry used in the micromagnetic simulations. (a) Spin waves propagate in a Co-Fe magnonic conduit of thickness t = 30 nm and width w = 1 μm. The external magnetic field H_{ext} is applied at a small tilt angle β (in the xz-plane) relative to the normal to the sample plane. (b) and (c) are schematics illustrating the relation between the vectors of the external magnetic field H_{ext}, the demagnetising field H_{demag}, and the effective field H_{eff} , to which the magnetisation M is parallel. (d) The wavenumber of crossing points as a function of the tilt angle β. (e) The lattice-period-dependent magnon excitation efficiency (red line) and the detection efficiency of the antenna (black line).

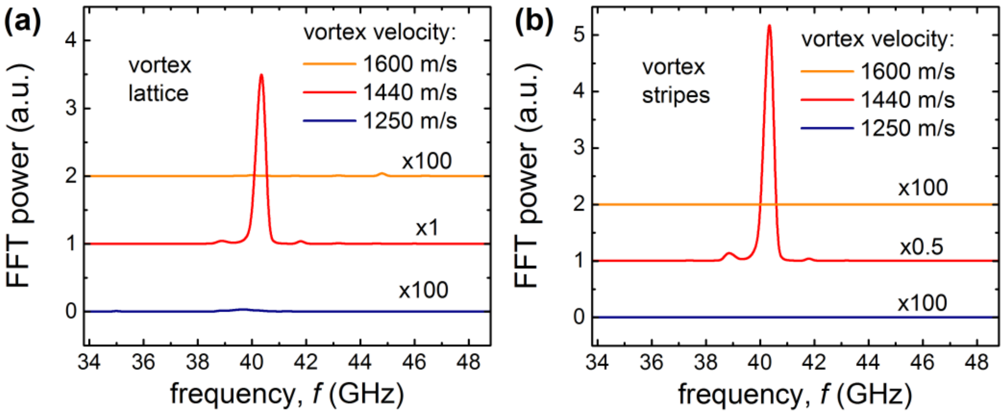

Supplementary Fig. 5. Simulated spin-wave spectra in the detection area. Simulated spin-wave spectra in the detection area for moving vortices ordered in a hexagonal lattice (a) and a stripe-like periodic pattern (b), with multiplication factors, as indicated.

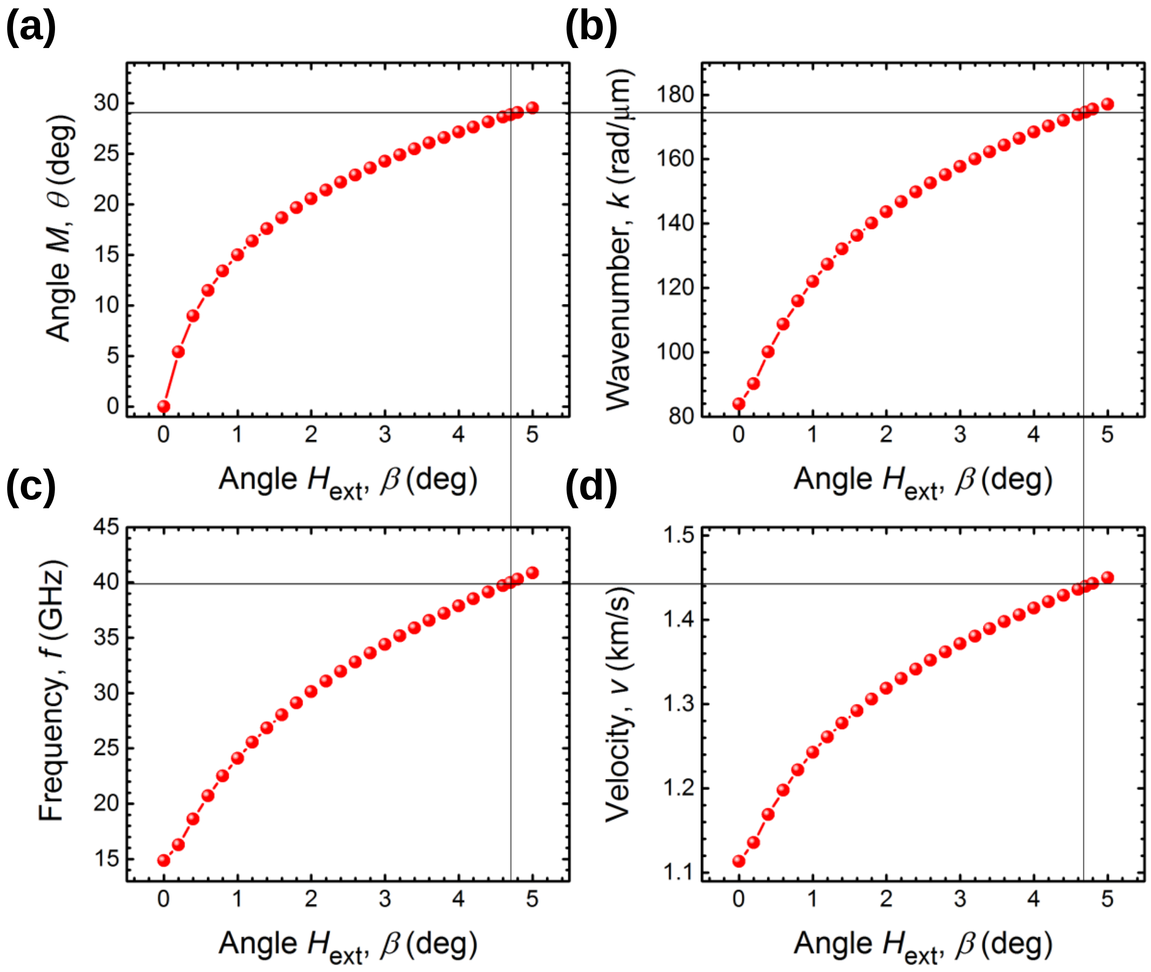

Supplementary Fig. 6. Magnonic parameters at the magnon generation condition. Dependences of the magnonic parameters at the magnon generation condition on the angle β between the external magnetic field H_{ext} and the z-axis for the 30 nm-thick Co-Fe spin-wave conduit with the best fit parameters of M_s = 1450 kA/m saturation magnetisation and A = 15 pJ/m the exchange stiffness, at H_{ext} = 1.85 T: (a) angle θ between the direction of the magnetisation M and the z-axis, (b) spin-wave wavenumber k, (c) spin-wave frequency f, and (d) spin-wave velocity v.

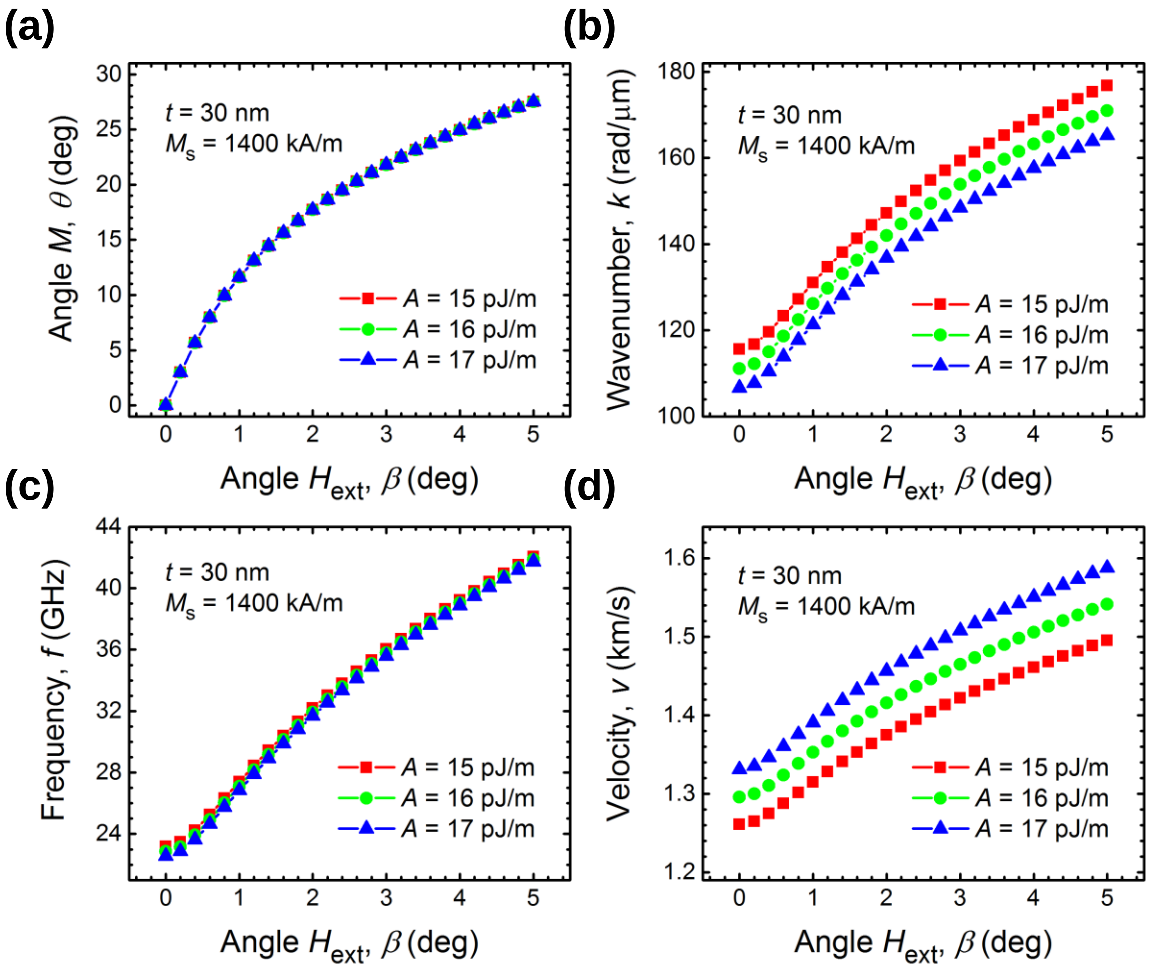

Supplementary Fig. 7. Dependence of the Co-Fe magnonic parameters on the exchange stiffness. Influence of the exchange stiffness A of the Co-Fe spin-wave conduit with the thickness t = 30 nm and the saturation magnetisation M_s = 1400 kA/m at H_{ext} = 1.85 T on (a) the angle θ between the direction of the magnetisation M and the z-axis, (b) the spin-wave wavenumber k, (c) the spin-wave frequency f, and (d) the spin-wave velocity v at the magnon generation condition as a function of the angle β between the applied magnetic field H_{ext} and the z-axis.

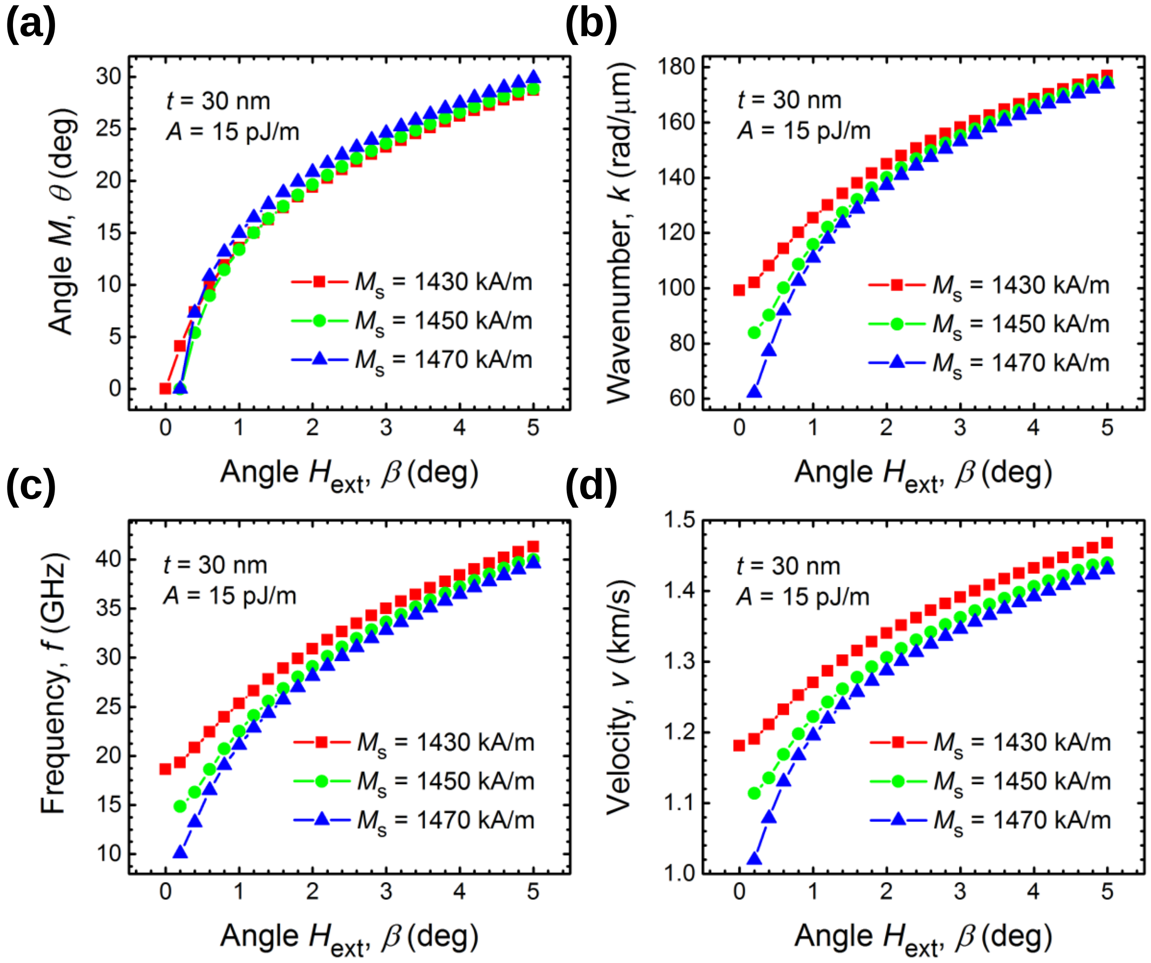

Supplementary Fig. 8. Dependence of the Co-Fe magnonic parameters on the saturation magnetisation. Influence of the saturation magnetisation M_s of the Co-Fe spin-wave conduit with the thickness t = 30 nm and the exchange stiffness A = 15 pJ/m at H_{ext} = 1.85 T on (a) the angle θ between the direction of the magnetisation M and the z-axis, (b) the spin-wave wavenumber k, (c) the spin-wave frequency f, and (d) the spin-wave velocity v at the magnon generation condition as a function of the angle β between the applied magnetic field H_{ext} and the z-axis.

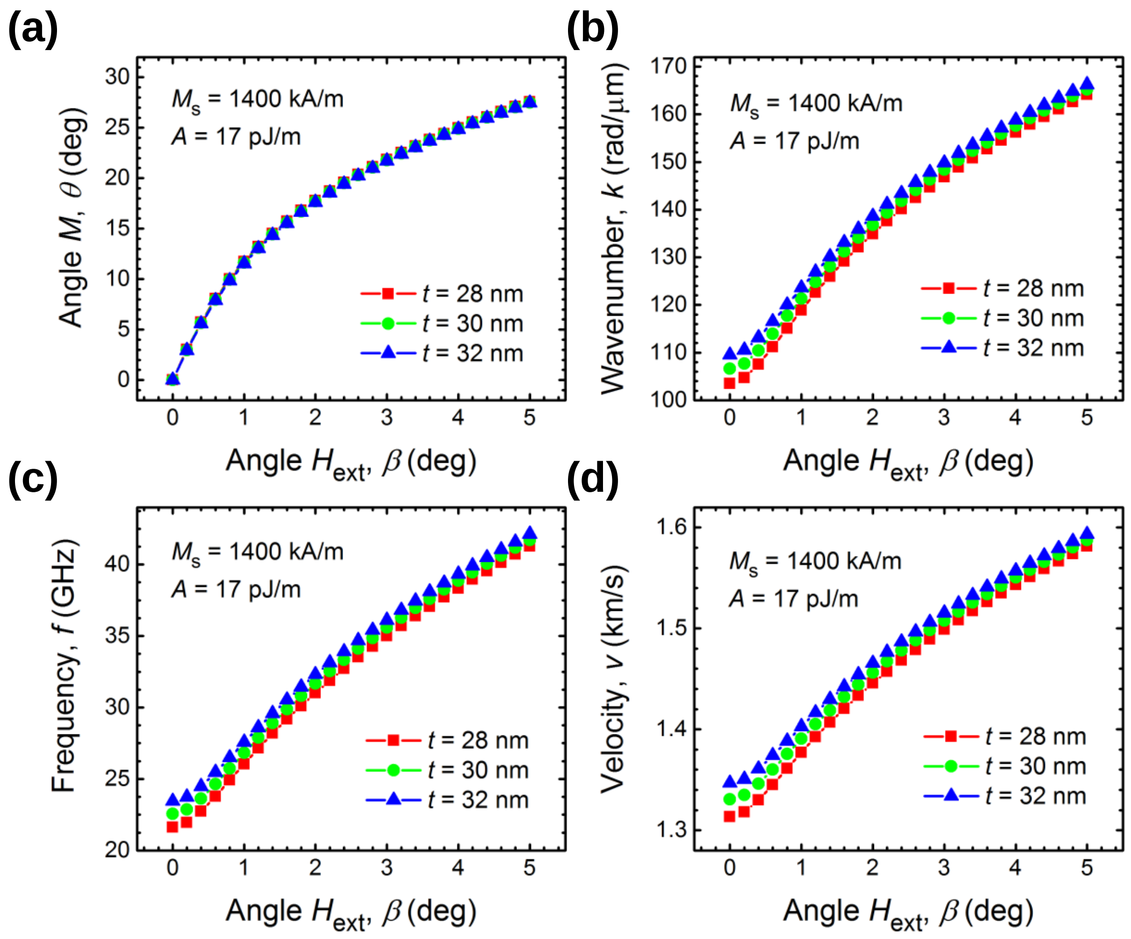

Supplementary Fig. 9. Dependence of the Co-Fe magnonic parameters on the conduit thickness. Influence of the saturation magnetisation M_s of the Co-Fe spin-wave conduit with the thickness t = 30 nm and the exchange stiffness A = 15 pJ/m at H_{ext} = 1.85 T on (a) the angle θ between the direction of the magnetisation M and the z-axis, (b) the spin-wave wavenumber k, (c) the spin- wave frequency f, and (d) the spin-wave velocity v at the magnon generation condition as a function of the angle β between the applied magnetic field H_{ext} and the z-axis.

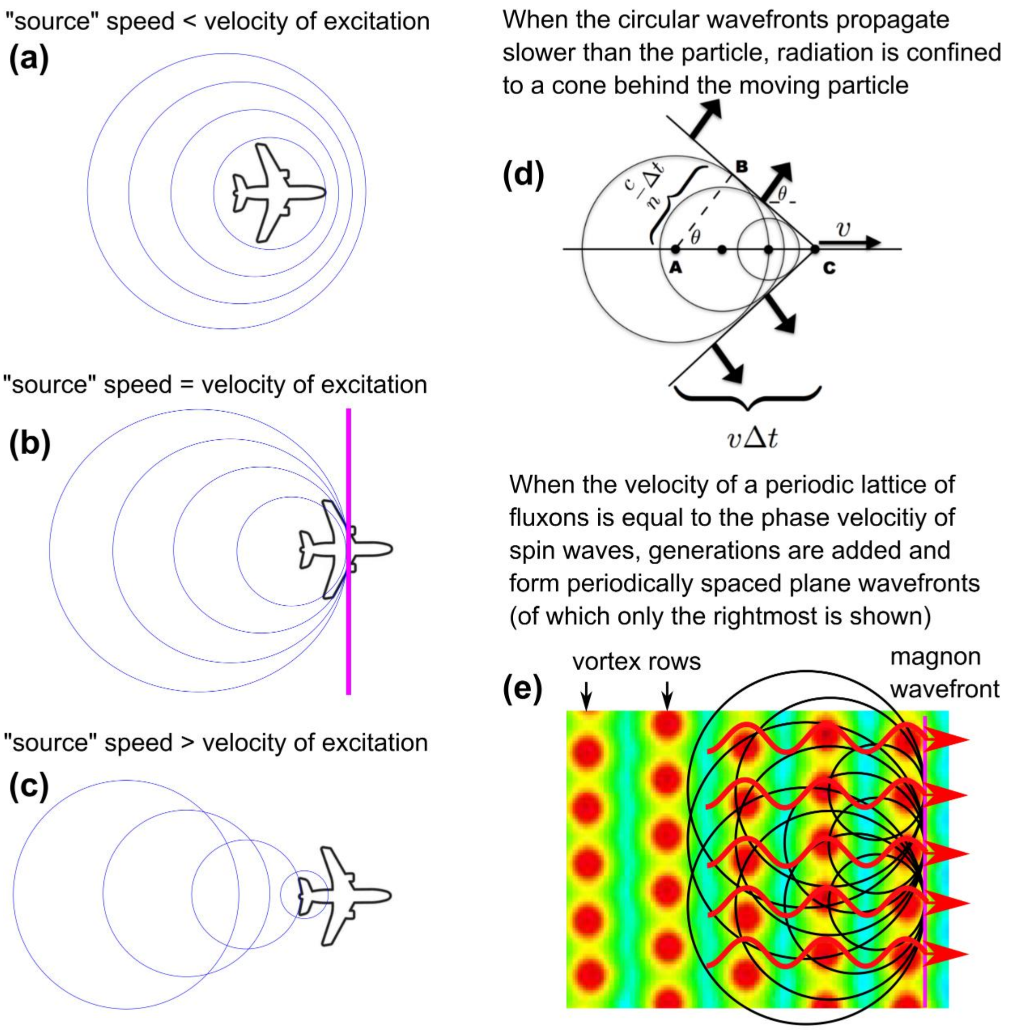

Supplementary Fig. 10. Relation between the Cherenkov effect for single and multiple periodically arranged moving particles. Cherenkov effect in aerodynamics: (a) subsonic velocities, no generation, but the Doppler effect is visible; (b) breaking the sound barrier and the Cherenkov generation at the resonance condition; (c) the Mach cone in the supersonic regime. The non-zero angle with respect to the motion of a charged particle only appears in the regime when the velocity of the moving particle is larger than the velocity of the generated wave. In "conventional" Cherenkov effect (d), the angle θ between the direction of propagation of the particle and the electromagnetic wave is given by $\cos\theta = c/(nv)$, where c/n is the speed of light in the medium, n is the refractive index of the medium, and v is the particle velocity. θ is very sharp when the particle moves much faster than c/n in the medium (e), and $\theta = 0$ for $v = c/n$ for the magnon generation condition in panels (b) and (e). In our studies of the generation of spin waves by a moving lattice of vortices (e), the generation of waves occurs not in 3D space but in an effectively 2D magnonic conduit where the wavefront is characterised by a straight line with $\theta = 0$. The relation to the "conventional" Cherenkov effect reads: $c/n \rightarrow v_{SW}$, $v \rightarrow v_{VL}$, $\theta \rightarrow 0$, single particle $\rightarrow N$ particles (in the general case).

Captions to Supplementary Videos

Video 1.

Spatiotemporal evolution of the magnetisation component m_x in the Co-Fe magnonic waveguide upon as the vortex lattice moves with velocity v_{VL} = 1250 m/s in the Nb-C superconductor (Sub-magnonic regime).

Video 2.

Spatiotemporal evolution of the magnetisation component m_x in the Co-Fe magnonic waveguide upon as the vortex lattice moves with velocity v_{VL} = 1440 m/s in the Nb-C superconductor (Magnonic regime I).

Video 3.

Spatiotemporal evolution of the magnetisation component m_x in the Co-Fe magnonic waveguide upon as the vortex lattice moves with velocity v_{VL} = 1600 m/s in the Nb-C superconductor (Magnonic regime II).

Video 4.

Spatiotemporal evolution of the superconducting order parameter in the bare Nb-C superconducting strip at the transport current I = 0.11 I_{dep} (depairing current of the superconductor).

Video 5.

Spatiotemporal evolution of the superconducting order parameter in the bare Nb-C superconducting strip at the transport current I = 0.12 $I_{dep.}$

Video 6.

Spatiotemporal evolution of the superconducting order parameter in the bare Nb-C superconducting strip at the transport current I = 0.13 $I_{dep.}$

Video 7.

Spatiotemporal evolution of the superconducting order parameter for the assumed hexagonal lattice of vortices in the Nb-C superconducting strip overlaid by the Co-Fe magnonic conduit at the transport current I = 0.11 $I_{dep.}$

Video 8.

Spatiotemporal evolution of the superconducting order parameter for the assumed hexagonal lattice of vortices in the Nb-C superconducting strip overlaid by the Co-Fe magnonic conduit at the transport current I = 0.12 $I_{dep.}$

Video 9.

Spatiotemporal evolution of the superconducting order parameter for the assumed hexagonal lattice of vortices in the Nb-C superconducting strip overlaid by the Co-Fe magnonic conduit at the transport current $I = 0.13\ I_{dep.}$

Video 10.

Spatiotemporal evolution of the superconducting order parameter for the assumed stripe-like periodic arrangement of vortices in the Nb-C superconducting strip overlaid by the Co-Fe magnonic conduit at the transport current $I = 0.11\ I_{dep.}$

Video 11.

Spatiotemporal evolution of the superconducting order parameter for the assumed stripe-like periodic arrangement of vortices in the Nb-C superconducting strip overlaid by the Co-Fe magnonic conduit at the transport current $I = 0.12\ I_{dep.}$

Video 12.

Spatiotemporal evolution of the superconducting order parameter for the assumed stripe-like periodic arrangement of vortices in the Nb-C superconducting strip overlaid by the Co-Fe magnonic conduit at the transport current $I = 0.13\ I_{dep.}$

Supplementary References

[1] B. A. Kalinikos, A. N. Slavin, J. Phys. C 19, 7013 (1986).
[2] D. Y. Vodolazov, Phys. Rev. Appl. 7, 034014 (2017).
[3] D. Y. Vodolazov, Supercond. Sci. Technol. 32, 115013 (2019).
[4] K. S. Tikhonov et al., Annals of Physics 417, 168101 (2020).
[5] K. E. Gray, Nonequilibrium Superconductivity, Phonons, and Kapitza Boundaries Plenum Press / Springer US, New York, NY, 1981, ISBN 978-0-306-40720-8.
